# 210 Preliminary Validation of the Arabic Global Neuropsychological Assessment (GNA) – ERRATUM

**DOI:** 10.1017/cts.2023.554

**Published:** 2023-06-14

**Authors:** John Gillies, Lauren Olson, Riam Almukhtar, Andy Strohmeier, Kinga Szigeti, David Schretlen, Renee Cadzow, Ralph Benedict

The above abstract was published with the incorrect title. The title should be as follows:

**Preliminary Validation of the Arabic Global Neuropsychological Assessment (GNA)**

Additionally, John Gillies should have been listed as an author.

The original abstract has been corrected online to rectify these errors.
